# 1266. Refining Antimicrobial Stewardship by De-escalating Vancomycin Using MRSA (*Methicillin Resistant Staphylococcus aureus*) Nasal Screening in a Community Hospital Setting

**DOI:** 10.1093/ofid/ofad500.1106

**Published:** 2023-11-27

**Authors:** Ruta Shah, Caroline Sorell, Monika Onussiet

**Affiliations:** MGB-Salem Hospital, Salem, Massachusetts; MGB-Salem Hospital, Salem, Massachusetts; MGB-Salem Hospital, Salem, Massachusetts

## Abstract

**Background:**

Our institution has prioritized appropriate use of anti-Methicillin-resistant Staphylococcus aureus (MRSA) drugs such as vancomycin. The overuse of anti-MRSA drugs contributes to drug toxicities and development of resistant organisms such as vancomycin*-*resistant enterococcus (VRE). Recent studies show that MRSA nasal surveillance screening has a high negative predictive value of 96.5% in all types of pneumonia. At our community hospital a decentralized pharmacist-driven MRSA nares screening protocol was novel. We report the results of implementing a MRSA nares screening protocol for vancomycin de-escalation in pneumonia patients.

**Figure:**
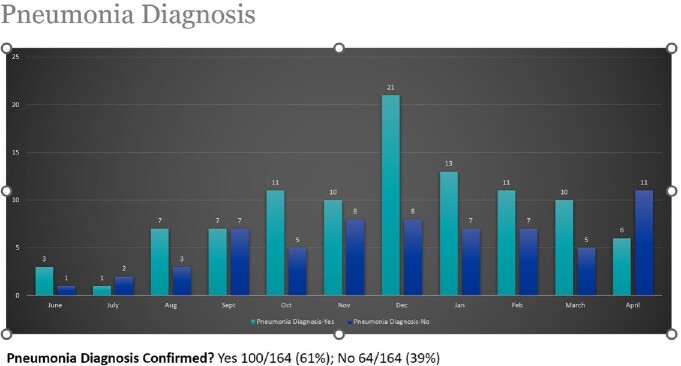

**Methods:**

A decentralized pharmacist reviewed patients with a diagnosis of pneumonia and prescribed vancomycin. The pharmacist ensured that a culture-based MRSA nares screen was performed within 48 hours of admission, if not already ordered. If the nares screen returned negative and respiratory cultures did not grow MRSA at 48 hours, the decentralized pharmacist contacted the medical team to recommend discontinuing vancomycin. Reasons for exclusion included a positive respiratory culture for MRSA within the prior 7 days, septic shock, alternative site of MRSA infection, and greater than 48 hours of anti-MRSA therapy at time of swab collection.

**Figure:**
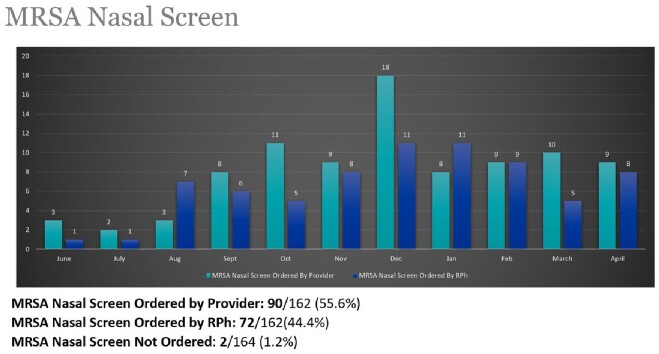

**Results:**

164 patients were reviewed by pharmacy between 6/16/22 and 4/25/23. 98% of MRSA nares screening had been performed within 48 hours of admission. MRSA screen was ordered by the decentralized pharmacist in 44% of patients. 89% of patients screened were negative for MRSA and reviewed for de-escalation of vancomycin. In 40% of patients, vancomycin was discontinued after recommendations by the decentralized pharmacist, with 95% of the recommendations accepted by the primary hospitalist physician. 37% of patients' vancomycin was discontinued by the attending physician. Overall, 77% of patients with negative MRSA nasal screen results were successfully de-escalated.

**Figure:**
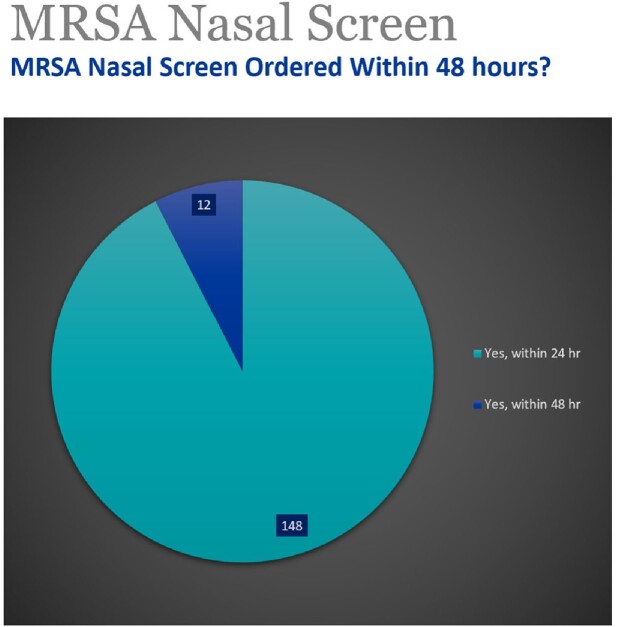

**Figure:**
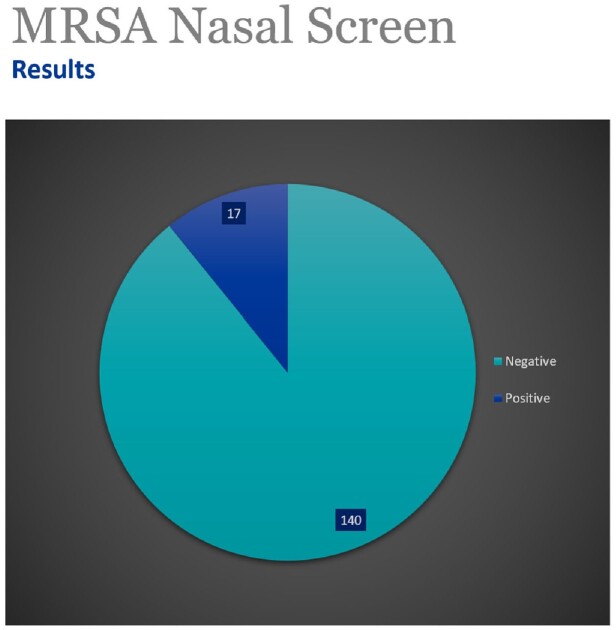

**Figure:**
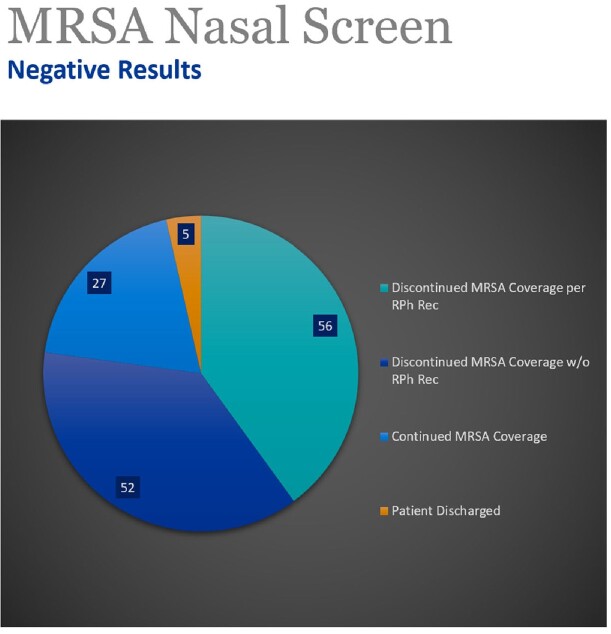

**Conclusion:**

Our data highlights the effectiveness of close physician-pharmacy collaboration. Most nares cultures were negative for MRSA allowing for de-escalation of antibiotics. Pharmacist recommendations were accepted majority of the time. Given the data, we plan to apply this approach for other ongoing stewardship initiatives.

**Disclosures:**

**All Authors**: No reported disclosures

